# Non-linear association of plasma level of high-density lipoprotein cholesterol with endobronchial biopsy bleeding in patients with lung cancer

**DOI:** 10.1186/s12944-019-0966-y

**Published:** 2019-01-18

**Authors:** Saibin Wang, Jingcheng Zhang, Xiaodong Lu

**Affiliations:** 1 Department of Respiratory Medicine, Jinhua Municipal Central Hospital, No. 365, East Renmin Road, Jinhua, Zhejiang Province, 321000 China; 2 Department of Hematology, Jinhua Municipal Central Hospital, No. 365, East Renmin Road, Jinhua, Zhejiang Province, 321000 China; 3 Department of Laboratory Medicine, Jinhua Municipal Central Hospital, No. 365, East Renmin Road, Jinhua, Zhejiang Province, 321000 China

**Keywords:** High-density lipoprotein, Lung cancer, Endobronchial biopsy, Bleeding

## Abstract

**Background:**

Despite a large body of studies have demonstrated the multifaceted behavior of high-density lipoproteins (HDLs) in several physiological and pathological processes, the levels of plasma HDL-cholesterol (HDL-C) that may be associated with endobronchial biopsy (EBB)-related bleeding have never been examined.

**Methods:**

We conducted a single-center retrospective cohort study of 628 consecutive patients with primary lung cancer who had undergone EBB at a large tertiary hospital between January 2014 and February 2018. Patients were divided into the bleeding group and the non-bleeding group according to the bronchoscopy report. The association between HDL-C levels and EBB-induced bleeding was evaluated using the LASSO regression analysis, multiple regression analysis and smooth curve fitting adjusted for potential confounders.

**Results:**

There was an inverse association of plasma HDL-C concentration with the incidence of EBB-induced bleeding as assessed by univariate analysis (*P* < 0.05). However, in piecewise linear regression analysis, a non-linear relationship with threshold saturation effects was observed between plasma HDL-C concentrations and EBB-induced bleeding. The incidence of EBB-induced bleeding decreased with HDL-C concentrations from 1.5 mmol/L up to 2.0 mmol/L (adjusted OR, 0.39; 95% CI, 0.20–0.74), but increased with HDL-C levels above the inflection point (HDL-C = 2.0 mmol/L).

**Conclusions:**

There was a non-linear association between plasma HDL-C concentrations and the risk of EBB-induced bleeding in patients with lung cancer. The plasma level of HDL-C above 2.0 mmol/L or below 1.5 mmol/L may increase the risk of EBB-induced bleeding.

## Background

Bronchoscopy is often required in patients with lung cancer, especially in their histopathological diagnosis [1]. However, worthy of note, biopsy-induced bleeding is frequently encountered during bronchoscopy, and massive bleeding in the airway could be life-threatening [2, 3]. Endobronchial biopsy (EBB), one of the most widely used transbronchial biopsy modalities, has been used in the diagnosis of pulmonary disease for more than 40 years [4]. Reportedly, the incidence of EBB-induced bleeding is relatively high, even over 30.0% in patients with lung cancer [5].

With regard to risk factors for bleeding during bronchoscopy, mechanical ventilation, immunosuppressive state, thrombocytopenia or anti-platelet therapy, anti-coagulant drugs use, liver and kidney disorders, heart function failure, and severe pulmonary arterial hypertension were proposed by several studies [6–8]. Nevertheless, whether these factors are in reality associated with bleeding during EBB, remain controversial [9, 10]. On the other hand, most individuals who are subjected to EBB do not have the aforementioned factors in clinical practice.

High-density lipoproteins (HDLs), traditionally known for its multiple protective effects on cardiovascular diseases, such as anti-atherosclerotic, anti-oxidative, anti-inflammatory and immune-modulating [11, 12], has been found to be associated with some hemorrhagic diseases in recent years [13–15]. Since factors affecting the risk of EBB-induced bleeding remain unclear, we hypothesized that plasma HDL-C may be associated with EBB-induced bleeding and had the potential to be a biomarker for EBB-induced bleeding. Therefore, we conducted this study to explore the association of plasma HDL-C with EBB-induced bleeding risk in patients with lung cancer.

## Methods

### Study population

This retrospective cohort study was based on 628 consecutive lung cancer patients who underwent EBB between January 2014 and February 2018 at the Jinhua Hospital of Zhejiang University, Jinhua, China. All included patients met the following criteria: a. age ≧18 years; b. local exophytic lesions located in tracheobronchial; and c. pathological diagnosis of primary lung cancer. Patients with any of the following factor were excluded, including active bleeding, platelets count < 50 × 10^3^/μl or continuous anti-platelet therapy, continuous anti-coagulant drug use, severe liver or kidney disorders, heart function failure, mechanical ventilation, and immunosuppressive state.

Patients were divided into two groups based on the biopsy results. Specifically, patients received hemostasis maneuvers during EBB were classified to the bleeding group (*n* = 232); whereas those who did not need hemostasis maneuvers or did not experience hemorrhage during EBB were classified to the non-bleeding group (*n* = 396). Hemostasis maneuvers during EBB included endobronchial perfusion using 4 °C physiological saline or/and diluted adrenalin (1:10000), and argon plasma coagulation application.

The following characteristics of the study participants were collected: age, sex, smoking (yes or no), lesions location (the trachea, left main bronchi, right main bronchi, and right middle bronchus were classified as central airways; whereas the left lobar bronchi and the right lobar bronchi were classified as peripheral bronchi), histological types of lung cancer (adenocarcinoma, squamous cell carcinoma, small-cell lung carcinoma, and the other types), TNM stage (stage I and II was considered as early stage; stage III and IV was considered as advanced stage), and comorbidities, including chronic obstructive pulmonary disease (COPD), coronary heart disease (CHD), hypertension, and diabetes mellitus. The following blood tests (performed on admission, prior to EBB) were collected: high density lipoprotein cholesterol (HDL-C), low density lipoprotein cholesterol (LDL-C), total cholesterol (TC), triglyceride, apolipoprotein E, apolipoprotein B, white blood cell counts, C-reactive protein (CRP), neutrophil counts, hemoglobin, platelet counts, prothrombin time (PT), activated partial thromboplastin time (APTT), alanine aminotransferase (ALT), and aspartate aminotransferase (AST).

This study was approved by the ethics committee of Jinhua Hospital of Zhejiang University, Jinhua, China. All patients’ information was anonymous preceding the analysis, and the requirement for informed consent, therefore, was waived.

### Biopsy procedures

All procedures were performed under general anesthesia. Propofol was used for induction (1.0 mg/kg) and maintenance (3.0–6.0 mg/kg/h), and remifentanil (5.0–10.0 μg/kg/h) was used for sedation and analgesia. During bronchoscopy, a laryngeal mask airway (Well Lead Medical Co., Ltd., Guangzhou, China) was used, and patients underwent transorally bronchoscopy. Bronchoscopy (BF-1 T60, Olympus Corp., Tokyo, Japan) procedures were performed on all patients by two experienced bronchoscopists. Three to five biopsies were generally performed at the same location of the endobronchial lesion by forceps biopsies [16]; however, in a small number of patients, we performed only one biopsy because lesions bled significantly following the first biopsy attempt.

### Statistical analysis

Patients’ baseline characteristics were summarized. Age and blood test values were presented as median (Q1-Q3), and categorical variables were presented as a number and percentage. Unpaired *t*-tests (normal distribution) or Kruskal-Wallis rank sum test (non-normal distribution), Pearson chi-squared tests or the Fisher’s exact, were tested between two groups for comparison, when appropriate. The least absolute shrinkage and selection operator (LASSO) regression method was employed to assess the strength of association between plasma HDL-C and EBB-induced bleeding. Multiple regression analysis was used to estimate the independent relationship between HDL-C levels and EBB-induced bleeding risk, with and without adjustment for potential confounders. We also used piecewise linear regression to test the threshold effect of HDL-C on EBB-induced bleeding together with a smoothing function. In this study, the adjusted criteria I included variables producing a change in the regression coefficient greater than 10% after introduction into the basic model or removing from the complete model (smoking, histological types, stage, triglyceride, PT, APTT and CRP); the screening criteria II included variables in criteria I, the regression coefficient of co-variable to dependent variable of *P* < 0.1 (lesions location and neutrophils), and judged by clinical significance (sex, age, and apolipoprotein E). All analyses were performed using R software (The R Foundation; https://www.r-project.org/). A value of *P* < 0.05 was considered statistically significant.

## Results

Of 628 consecutive patients, 232 (36.9, 95% confidence interval [CI], 33.2–40.7%) received hemostasis maneuvers following EBB (4 °C physiological saline or/and diluted adrenalin endobronchial perfusion, or argon plasma coagulation). No patient died of severe blood loss after a biopsy. Patients’ baseline characteristics and blood tests are shown in Table 1.

**Table 1:** Baseline characteristics and blood tests of the study participants

Characteristics	Values
Age (year), median (Q1-Q3)	65 (59–70)
Male, n (%)	487 (77.5)
Smoking, n (%)	394 (62.7)
Biopsy bleeding, n (%)	232 (36.9)
Location of lesion, n (%)	
Peripheral bronchi	548 (87.3)
Central airway	80 (12.7)
Stage, n (%)	
Early	343 (54.6)
Advanced	285 (45.4)
Histological types, n (%)	
Adenocarcinoma	171 (27.2)
Squamous cell carcinoma	313 (49.8)
SCLC	111 (17.7)
Others	33 (5.3)
Coexisting diseases, n (%)	
COPD	42 (6.7)
Hypertension	155 (24.7)
Diabetes	32 (5.1)
CHD	20 (3.2)
Blood tests, median (Q1-Q3)	
Triglyceride (mmol/L)	1.0 (0.8–1.4)
TC (mmol/L)	4.1 (3.5–4.8)
HDL-C (mmol/L)	1.1 (0.9–1.3)
LDL-C (mmol/L)	2.8 (2.3–3.3)
Apo E (mg/dL)	3.6 (2.8–4.6)
Apo B (g/L)	1.0 (0.8–1.2)
Homocysteine (μmol/L)	13.2 (10.7–16.2)
WBC (× 10^9^/L)	6.8 (5.5–8.6)
Neutrophils (×10^9^/L)	4.6 (3.5–6.4)
Hemoglobin (g/L)	128 (116–139)
Platelets (×10^9^/L)	224 (172–280)
CRP (mg/L)	7.7 (1.4–31.1)
PT (S)	13.0 (12.3–13.6)
APTT (S)	35.1 (31.8–38.5)
ALT (IU/L)	17.0 (12.0–25.0)
AST (IU/L)	23.0 (19.0–28.0)

*SCLC* Small-cell lung carcinoma; *COPD* Chronic obstructive pulmonary disease; *CHD* Coronary heart disease; *TC* Total cholesterol; *HDL-C* High density lipoprotein cholesterol; *LDL-C* Low density lipoprotein cholesterol; Apo, apolipoprotein; *WBC* White blood cell; *CRP* C-reactive protein; *PT* Prothrombin time; *APTT* Activated partial thromboplastin time; *ALT* Alanine aminotransferase; *AST* Aspartate aminotransferase

**Fig. 1:**
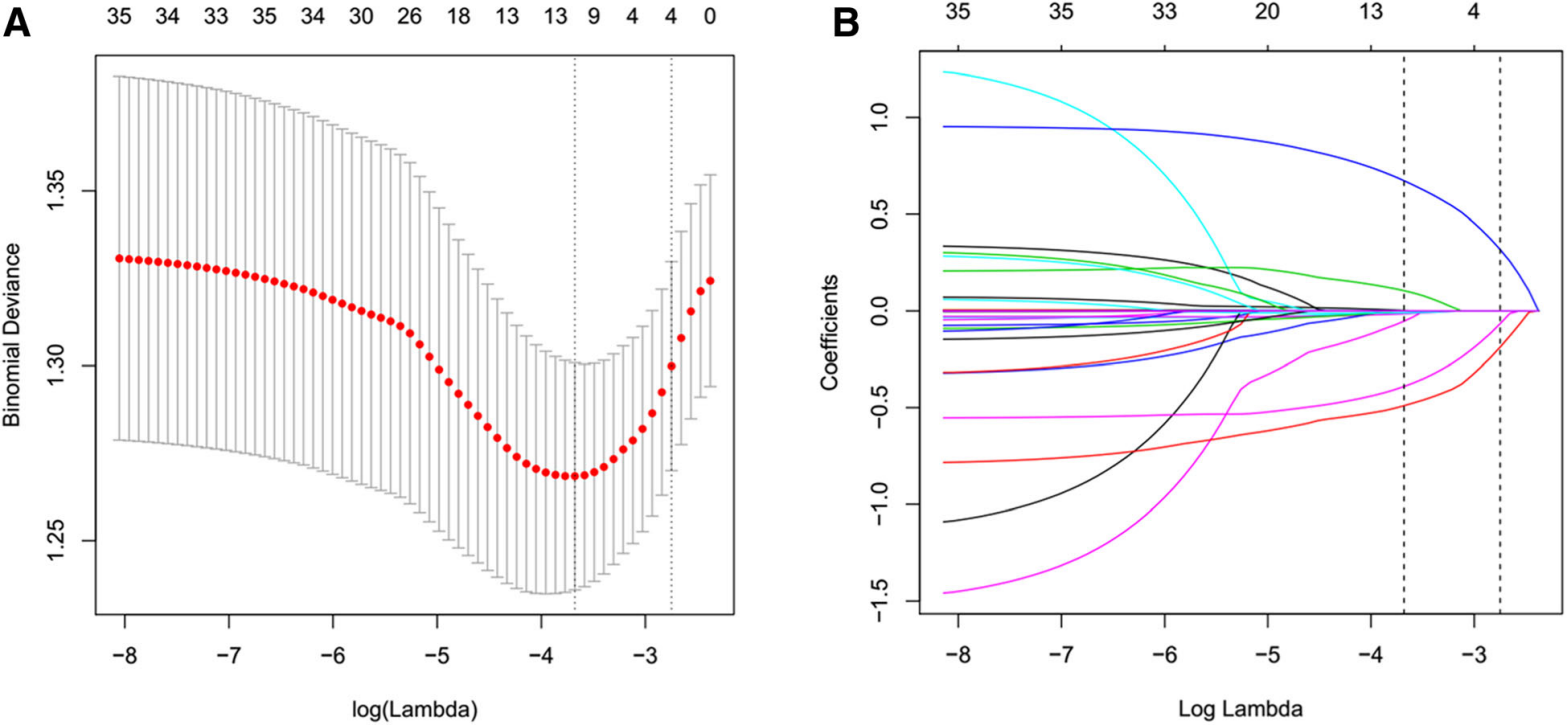
The strength of association between variables with EBB-induced bleeding in the LASSO regression method. **a** Tuning variable (lambda) selection using 10-fold cross-validation in the LASSO regression. Dotted vertical lines were drawn at the optimal values based on the minimum criteria (left dotted line) and the 1-SE criteria (right dotted line). **b** A coefficient profile plot was produced against the log (lambda) sequence. In the current study, variables were filtered according to the minimum criteria (left dotted line), where optimal lambda resulted in 7 nonzero coefficients, including HDL-C (− 0.0527), APTT (− 0.0146), neutrophils (0.0024), CRP (0.0015), lesion location (0.6728), TNM stage (− 0.3905) and histological types (− 0.4902). SE = standard error

In univariate analysis, the plasma HDL-C concentrations were lower in the bleeding group compared to those in the non-bleeding group (Table 2, *P* < 0.05). In addition, lesions location, histological types, TNM stage of cancer, smoking, CRP, and APTT were associated with EBB-induced bleeding (Table 2). Based on non-zero coefficients in the LASSO regression analysis (Fig. 1), seven variables were filtered. These variables (coefficient) were HDL-C (− 0.0527), APTT (− 0.0146), neutrophils (0.0024), CRP (0.0015), lesion location (0.6728), TNM stage (− 0.3905) and histological types (− 0.4902), among which the absolute value of coefficient of HDL-C was the maximum in all blood variables, suggesting that HDL-C has a stronger association with EBB-induced bleeding.

**Table 2:** Univariate analysis of possible influencing factors of the risk of EBB-induced bleeding

Variables	EBB-induced Bleeding	
	OR (95% CI)	*P* value
Age (year)	1.01 (0.99, 1.03)	0.2029
Sex		
Female	Ref.	
Man	1.33 (0.89, 1.98)	0.1609
Smoking		
No	Ref.	
Yes	1.45 (1.03, 2.04)	0.0337
Location of lesion		
Central airway	Ref.	
Peripheral bronchi	0.32 (0.20, 0.51)	< 0.0001
Stage		
Early	Ref.	
Advanced	1.87 (1.35, 2.60)	0.0002
Histological type		
Adenocarcinoma	Ref.	
Squamous cell carcinoma	2.63 (1.73, 4.01)	< 0.0001
SCLC	2.14 (1.27, 3.61)	0.0043
Others	2.20 (1.00, 4.82)	0.0488
COPD		
No	Ref.	
Yes	1.05 (0.55, 2.01)	0.8727
Hypertension		
No	Ref.	
Yes	1.11 (0.76, 1.61)	0.5995
Diabetes		
No	Ref.	
Yes	0.89 (0.42, 1.88)	0.7575
CHD		
No	Ref.	
Yes	1.14 (0.46, 2.84)	0.7736
Triglyceride (mmol/L)	0.80 (0.62, 1.04)	0.1018
TC (mmol/L)	0.93 (0.78, 1.11)	0.4113
HDL-C (mmol/L)	0.56 (0.34, 0.94)	0.0270
LDL-C (mmol/L)	0.89 (0.71, 1.11)	0.3031
Apo E (mg/dL)	0.91 (0.82, 1.02)	0.1153
Apo B (g/L)	0.80 (0.45, 1.42)	0.4421
Homocysteine (μmol/L)	1.00 (0.98, 1.02)	0.8840
WBC (×10^9^/L)	1.03 (0.98, 1.08)	0.2477
Neutrophils (×10^9^/L)	1.04 (0.99, 1.10)	0.0937
Hemoglobin (g/L)	1.00 (0.99, 1.01)	0.4135
Platelets (×10^9^/L)	1.00 (1.00, 1.00)	0.2392
CRP (mg/L)	1.01 (1.00, 1.01)	0.0173
PT (S)	0.93 (0.84, 1.02)	0.1220
APTT (S)	0.96 (0.94, 0.99)	0.0180
ALT (IU/L)	1.00 (0.99, 1.01)	0.8181
AST (IU/L)	1.00 (0.99, 1.01)	0.9365

*EBB* endobronchial biopsy; *SCLC* small-cell lung carcinoma; *COPD* Chronic obstructive pulmonary disease; *CHD* coronary heart disease; *TC* total cholesterol; *HDL-C* high density lipoprotein cholesterol; *LDL-C* low density lipoprotein cholesterol; Apo, apolipoprotein; *WBC* white blood cell; *CRP* C-reactive protein; *PT* prothrombin time; *APTT* activated partial thromboplastin time; *ALT* alanine aminotransferase; *AST* aspartate aminotransferase

The association between plasma HDL-C and EBB-induced bleeding risk was shown in Table 3 after adjusting for smoking, histological type, stage of cancer, triglyceride, PT, APTT and CRP (adjust criterion I), or after adjusting for sex, age, smoking, location of lesion, histological type, stage of cancer, triglyceride, apolipoprotein E, PT, APTT, neutrophils and CRP (adjust criterion II). In piecewise analysis, we found that middle concentrations of HDL-C (1.5–2.0 mmol/L) associated with a decreased risk of EBB-induced bleeding when compared to lower concentrations (< 1.5 mmol/L) (odds ratio [OR], 0.39; 95% CI, 0.20–0.74; *P* < 0.05). However, higher concentrations of HDL-C (≧ 2.0 mmol/L) did not associate with a decreased incidence of EBB-induced bleeding (OR, 1.63; 95% CI, 0.51–5.22; *P* > 0.05).

**Table 3:** Multivariate regression analysis of HDL-C with the risk of EBB-induced bleeding

HDL-C (mmol/L)	EBB-induced bleeding OR (95% CI) *P*-value		
	Non-adjust	Adjust I^a^	Adjust II^b^
< 1.5	Ref.	Ref.	Ref.
1.5–2.0	0.35 (0.19, 0.64) 0.0007	0.38 (0.20, 0.71) 0.0026	0.39 (0.20, 0.74) 0.0042
≧2.0	1.55 (0.54, 4.48) 0.4187	1.95 (0.65, 5.88) 0.2339	1.63 (0.51, 5.22) 0.4107

^a^Adjust I adjust for: smoking, histological type, stage of cancer, triglyceride, PT, APTT and CRP

^b^Adjust II adjust for: sex, age, smoking, location of lesion, histological type, stage of cancer, triglyceride, apolipoprotein E, PT, APTT, neutrophils and CRP. EBB, endobronchial biopsy; HDL-C, high density lipoprotein cholesterol; PT, prothrombin time; APTT, activated partial thromboplastin time; CRP, C-reactive protein

**Fig. 2:**
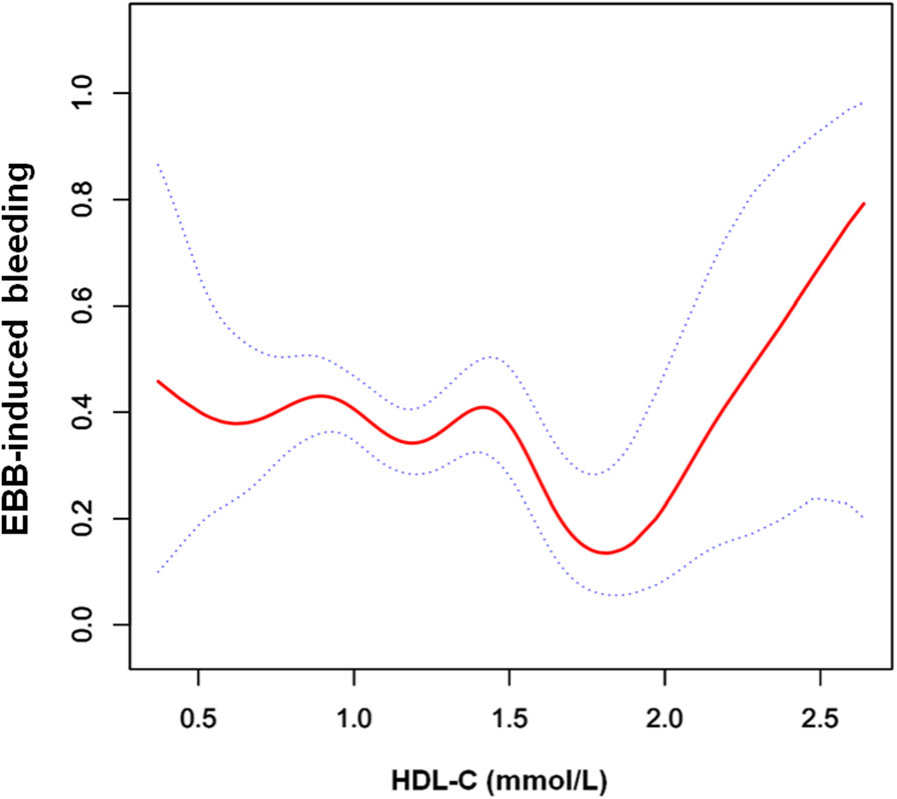
A non-linear relationship with threshold effect between plasma HDL-C concentrations and EBB-induced bleeding risk in the smooth curve fitting after adjusting the potential confounding factors (sex, age, smoking, location of lesion, histological type, stage of cancer, triglyceride, apolipoprotein E, PT, APTT, neutrophils and CRP) is shown in the figure. Dotted lines represent the upper and lower 95% confidence intervals. EBB = endobronchial biopsy; HDL-C = high density lipoprotein cholesterol; PT = prothrombin time; APTT = activated partial thromboplastin time; CRP = C-reactive protein

A non-linear relationship between plasma HDL-C levels and the risk of EBB-induced bleeding was observed in the smooth curve fitting (Fig. 2). Two threshold values (inflection point I = 1.4 mmol/L, and inflection point II = 1.9 mmol/L) were detected between plasma HDL-C levels and EBB-induced bleeding risk in two-piecewise linear regression analysis (Table 4).

**Table 4:** Threshold effect analysis of HDL-C on EBB-induced bleeding using two-piecewise linear regression

Inflection point of HDL-C (mmol/L)	EBB-induced Bleeding^a^	
	OR (95% CI)	*P* value
Inflection point I		
< 1.4	1.03 (0.42, 2.51)	0.9554
> 1.4	0.02 (0.00, 0.41)	0.0118
Inflection point II		
< 1.9	0.49 (0.25, 0.95)	0.0343
> 1.9	19.99 (0.85, 470.30)	0.0631

^a^Adjust for: sex, age, smoking, location of lesion, histological type, stage of cancer, triglyceride, apolipoprotein E, PT, APTT, neutrophils and CRP. EBB, endobronchial biopsy; HDL-C, high density lipoprotein cholesterol; PT, prothrombin time; APTT, activated partial thromboplastin time; CRP, C-reactive protein

## Discussion

Our findings showed a statistically significant non-linear association between HDL-C level and risk of bleeding during EBB. Specifically, in order to reduce the risk of EBB-induced bleeding, especially for massive bleeding, it may be helpful to maintain the concentrations of plasma HDL-C within 1.5–2.0 mmol/L prior to aggressive biopsies on endobronchial exophytic lesions in patients with lung cancer.

Bronchoscopy-related bleeding is a very common complication in clinical practice, especially when biopsies are performed. Of note, malignant lesions reportedly are more likely to bleed upon biopsy than benign mucosal lesions [17], and the incidence of massive hemorrhage increases following EBB [18]. In this scenario, a number of studies have been conducted and several risk factors that may be associated with bleeding during bronchoscopy have been proposed, such as immunosuppressive state, thrombocytopenia (< 50 × 10^3^/μl), anti-platelet or anti-coagulant drugs use, severe liver and kidney disorders, heart function failure, severe pulmonary arterial hypertension, and lung transplant [6–8]. However, most of the aforementioned factors remain conflicting or lack supporting evidence [9, 10]. To our knowledge, there is still no effective indicator or biomarker available for predicting bleeding risk during bronchoscopy in clinical practice.

HDLs are heterogeneous lipoproteins involve in multiple physiological and pathological processes in the human body [19]. During the last few decades, most studies conducted have demonstrated HDL-C as a protective factor in cardiovascular disease [19]. In recent year, several studies revealed that HDL-C was associated with the risk of hemorrhage in some disorders, especially in intracranial hemorrhage [13, 20]. In this regard, several studies have shown that the risk of intracerebral hemorrhage significantly increased with decreasing HDL-C concentrations [14, 15]. However, in a meta-analysis of 1,430,141 participants from 23 prospective studies, the inverse relationship between intracerebral hemorrhage and HDL-C was pointed out by researchers [13]. Despite the fact that the relationship between HDL-C and the risk of intracerebral hemorrhage remains controversial, these studies, at least, have indicated that HDL-C may be associated with the risk of bleeding in some disorders.

The current knowledge in the realm of the effect of HDL on coagulation and clotting cascade remains lacking. Adiponectin, an adipose-derived cytokine, may partially explain the effect of HDL on coagulation. Several studies have demonstrated that, both in vitro and in vivo, HDL associates strongly with adiponectin [21–23]. Reportedly, adiponectin could affect platelet hyperactivity, hypercoagulability, and hypofibrinolysis [24–26]. It plays an anticoagulant role by increasing the expression of plasminogen activator inhibitor (PAI)-I both in human and in animal models [27–30]. Therefore, the effect of HDL on coagulation may be mediated partially through the suppressive effect of adiponectin on PAI-I production. In addition, HDL could modulate the function of vascular smooth muscle and platelets [31, 32].

The present study found that plasma HDL-C concentrations strongly associated with the incidence of EBB-induced bleeding in LASSO regression analysis, which is considered to surpass the approach of identifying variables based on the strength of their univariable association with the outcome, especially when there are a bunch of variables [33, 34]. This association still strengthened after controlling for potentially confounding variables (adjust I controlling for: smoking, histological type, stage of cancer, triglyceride, PT, APTT and CRP; adjust II controlling for: sex, age, smoking, location of lesion, histological type, stage of cancer, triglyceride, apolipoprotein E, PT, APTT, neutrophils and CRP) in in multiple regression analysis. There was a non-linear association between plasma HDL-C levels and EBB-induced bleeding risk. We further uncovered a piecewise effect of HDL-C concentrations against the risk of EBB-induced bleeding. Between 1.5–2.0 mmol/L, HDL-C was associated with lower bleeding risk, whereas higher (> 2.0 mmol/L) or lower (< 1.5 mmol/L) concentrations of HDL-C were associated with increased EBB-induced bleeding risk. This finding may increase our understanding of the novel role of HDL-C.

The current study is the first to reveal the relationship between plasma HDL-C levels and EBB-induced bleeding risk during bronchoscopy. However, several limitations of the present study are worth noting. First, although we had adjusted for potentially confounding factors, this study was inevitably subject to the limitations of the use of observational data. Second, a quantitative measurement of the volume of EBB-induced bleeding in our study was unavailable. It remains challenging to accurately measure bleeding during bronchoscopy [35]. Therefore, the grouping of biopsy bleeding may not be accurate in this study because we divided participants into a bleeding group or a non-bleeding group based only on whether they received hemostasis during EBB. Third, we only collected HDL-C concentrations at admission, and repeated measurements were unavailable in the retrospective data. Therefore, further validation in prospective studies is warranted. Despite these limitations, this study has notable clinical implications since no effective indicator for EBB-induced bleeding has been found to date.

## Conclusions

This study revealed that plasma HDL-C levels are associated with EBB-induced bleeding risk in a non-linear pattern in patients with lung cancer, and the relative safe concentrations of plasma HDL-C on EBB bleeding may be 1.5–2.0 mmol/L. This finding highlights the importance of HDL-C concentrations on EBB and may have implications for bleeding risk assessment and risk modification prior to EBB. Future studies are needed to fully evaluate the effect of HDL-C on EBB-induced bleeding and investigate the underlying mechanisms.

## Abbreviations

ALT: Alanine aminotransferase
APC: Argon plasma coagulation
APTT: Activated partial thromboplastin time
AST: Aspartate aminotransferase
CHD: Coronary heart disease
CI: Confidence interval
COPD: Chronic obstructive pulmonary disease
CRP: C-reactive protein
EBB: Endobronchial biopsy
HDL-C: High density lipoprotein cholesterol
LASSO: The least absolute shrinkage and selection operator
LDL-C: Low density lipoprotein cholesterol
OR: Odds ratio
PAI: Plasminogen activator inhibitor
PT: Prothrombin time
TC: Total cholesterol
WBC: White blood cell
